# Incidence of Severe Hypothermia and Its Impact on Postoperative Surgical Complications and Time Delay to Adjunct Treatments in Breast Surgery Cancer Patients: A Case-Controlled Study

**DOI:** 10.3390/jcm10163702

**Published:** 2021-08-20

**Authors:** Cyrus Motamed, Gregoire Weil, Chaima Dridi, Jean Louis Bourgain

**Affiliations:** 1 Department of Anesthesia, Gustaveroussy Cancer Campus, 94080 Villejuif, France; chaimadridi23@gmail.com (C.D.); bourgainjl@orange.fr (J.L.B.); 2 Service d’Anesthésie Reanimation, Centre Hospitalier Regional d’Orléans, 45067 Orléans, France; gregoire.weil@chr-orleans.fr

**Keywords:** postoperative hypothermia, wound infection, breast cancer surgery

## Abstract

Introduction: Unintended postoperative hypothermia frequently occurs upon arrival in the post anesthesia care unit (PACU). As part of our quality assurance program in anesthesia, we regularly monitor the incidence of this complication through our anesthesia information management system (AIMS). In this case-controlled retrospective study, our goal was to detect the incidence of unintended severe hypothermia in our breast surgery cancer patients, and subsequently to analyze the consequence of this complication in terms postoperative cutaneous infection, as well as its impact on further complementary treatment, such as radiotherapy and chemotherapy. Methods: This study was a retrospective analysis conducted through our AIMS system from 2015 through 2019, with extraction criteria based on year, type of surgery (breast), and temperature upon arrival in PACU. A tympanic temperature of less than 36 °C was considered to indicate hypothermia. Severe hypothermia was considered for patients having a temperature lower than 35.2 °C (hypothermic) (*n* = 80), who were paired using a propensity score analysis with a control group (normothermic) (*n* = 80) of other breast cancer surgery patients. Extracted data included time of surgery, sex, age, ASA status, and type and duration of the intervention. Results: The mean incidence of hypothermia was 21% from 2015 through 2019. The body mass index (BMI) was significantly lower in the hypothermia group before matching, 23.5 ± 4.1 vs. 26.4 ± 6.1 kg/m^2^ in normothermic patients (*p* < 0.05). The hypothermia group also had significantly fewer monitoring and active warming devices. No difference was noted for wound complications. Time to complementary chemotherapy and or radiotherapy did not differ between groups (52 ± 21 days in group hypothermia vs 49 ± 22 days in the control group). Conclusion: Severe intraoperative hypothermia remains an important quality assurance issue in our breast surgery cancer patients, but we could not detect any long-term effect of hypothermia.

## 1. Introduction

Perioperative hypothermia increases perioperative morbidity [1,2], including postoperative wound infections, that, because of their frequency and severity, are a significant risk for surgery patients [3]. The prevention of postoperative infectious complications depends on pre-incisional antibiotic prophylaxis and perioperative blood sugar control, as well as on maintenance of normothermia [3]. In addition to intraoperative warming, prewarming is also reported to significantly contribute to the maintenance of normothermia, decreasing the incidence of surgical site infection [4]. However, to be effective, prewarming should be performed adequately by respecting a minimum dedicated time of 15 minutes [5,6]. 

We previously reported an incidence of mild hypothermia in our institution as part of our quality assurance program in anesthesia [7,8]. However, we did not evaluate any other related complications specific to our surgical cancer patients. This quality assurance program largely relies on our Anesthesia Information Management System (AIMS), which permits the regular and easy monitoring of different quality assurance anesthesia indicators to improve our practice [8,9].

Oncological surgery can cause an alteration of the host’s immune defenses to favor the proliferation of cancer cells [10]. Many factors, including hypothermia, are involved in this perioperative immunomodulation [11]. The literature is clear on the mortality and morbidity induced by postoperative hypothermia [11]; however, the practical implications that hypothermia might have in routine clinical practice such as wound infection in cancer surgery or possible delays in further oncologic treatment of cancer patients, have not been sufficiently studied. 

The primary objective of the present study was to detect the trends and incidence of severe unintended hypothermia in breast cancer surgical patients. Our secondary objective was to assess the impact of hypothermia on postoperative surgical wound complications and, as well as possible time delays in complementary cancer care, including radio- and/or chemotherapy.

## 2. Methods

This retrospective, single-center, propensity-matched cohort study was carried out in the anesthesia department of the Gustave Roussy Cancer Institute in Villejuif, France from February to May 2020. The completely retrospective nature of the study allowed waiver of informed consent, since no change in practice was required with approval of our institutional review board on April 2021 to exploit our database for quality assurance purposes Avis n° 2021/32. Data were extracted from our AIMS system using the Centricity Anesthesia database GE (Barrington, IL, USA) and Crystal report Software V.12 2008 (COMBIT, Konstanz, Germany) as an interface. Our anesthesia database administrator (JLB) had several cycles of formal training in order to master the appropriate data extraction from the database with this software. 

In order to verify each broad extraction, a preliminary extraction of the same query through a very short period (3 days) was checked manually before extracting the targeted data for the broader period of time from 1 January 2015 to 31 December 2019. Data were cleaned of redundancy and missing data were calculated using Microsoft Excel 2010 spreadsheets (Microsoft, Redmond, WA, USA).

Patients were classified according to their temperature upon arrival in the PACU and separated into two categories of hypothermic (less than 36.5 °C) and normothermic (above 36.5 °C). Anesthesia protocols were at the discretion of anesthesiologists in charge, they always consisted of propofol IV bolus and remifentanil target-controlled infusion (RTCI) for the induction and were adjusted for maintenance in addition to inhalational anesthesia or propofol IV. Prevention of hypothermia consisted of prewarming, if possible, the use of a simple blanket, upper body blanket for air warming, esophageal probe for intraoperative monitoring tympanic probe, and electrical blanket in the PACU. If surgery was scheduled for less than an hour, forced air warming was not mandatory.

In the PACU active air warming, warming blanket, or simple blanket was used to obtain normothermia depending on the temperature at arrival. 

The “normothermic” control group was created from an exhaustive database of 6779 patients who underwent breast cancer surgery and had a temperature between 36.5 °C and 37 °C upon arrival in the PACU. One-by-one pairing was performed according to time of surgery, sex, age, and type and duration of the intervention.

The following data were extracted: Demographic data (age, sex, American Society of Anesthesiologists (ASA) physical status, height and weight), intraoperative events (including the type and the duration of surgery, duration of anesthesia, surgeon experience (fellow or staff), the percentage of procedures performed by junior surgeons in all breast surgery patients presence of temperature monitoring during intervention, use of a warming device Bair Hugger^®^ (3M, St. Paul, MN, USA) or simple blanket, administration of antibiotic prophylaxis, (antibiotic agent and dosage), and time between antibiotic administration and incision), and PACU data (including temperature upon arrival and length of stay). Postoperative surgical complications were scrutinized as follows: Skin necrosis (skin turning dark blue or black and eventually developing scabs and/or open wounds), cutaneous infection (skin becoming red, warm and inflamed with tendency to spread with or without flu like symptoms), lymphocele (collection of clear fluid which accumulates in the surgical cavity), and postoperative hematoma (collection of blood that forms under the skin’s surface).

Oncological treatments and possible delays were obtained by electronic consultation of each medical file. 

The collected data were anonymized and then recorded, analyzed, and described in an Excel spreadsheet (Microsoft, Redmond, WA, USA) and expressed as mean ± standard deviation for continuous variables and numbers and/or percentages for categorical and qualitative variables.

## 3. Statistical Analysis

To obtain a sufficient number of severe hypothermia patients for this study, group severe hypothermia was defined as patients having temperature upon arrival in PACU of ≤35.2 °C. We chose this threshold in order to extract approximately 100 patients from our AIMS. A minor change to this threshold such as 0.1 °C would significantly decrease or increase the number of patients. This initial sample size was chosen in order to obtain minimal wound complications as in the study of Kurz et al. [12].

Data are summarized as medians and interquartile ranges or as numbers and percentages. We realized a 1:1 nearest-neighbor propensity score matching without replacement with a propensity score estimated using logistic regression of the hypothermia status on the covariates (age, duration of anesthesia, ASA scores, and BMI). A caliper of 0.2 of the standard deviation of the PS logit was used [13]. Adequate balance was checked with standardized mean differences for the covariates below 0.1 [14].

The secondary outcomes were the incidence of postoperative wound complications and time delays to adjunct treatments. Univariable analyses were performed on the matched patients using the Wilcoxon signed rank test for paired continuous variables and the McNemar exact test for categorical variables. The incidence of the variables of the secondary outcome was compared between the propensity score-matched groups using logistic models. The odds of each covariate were characterized by odds ratios (ORs) from the logistic regression models. Comparison of the time elapsed between additional treatment was compared by a Kaplan–Meier survival analysis. All statistical analyses were performed using R Studio v.1.4.1106 (R Core Team, Vienna, Austria), including the “MatchIt” package. 

## 4. Results

A total of 6377 breast procedures were included in this extraction. The mean incidence of hypothermia (<36 °C) was 21% from 2015 to 2019, the incidence of severe hypothermia (<35 °C) was 1%. In total, 106 patients that had a temperature (<35.2 °C) were initially extracted as hypothermic patients. An equivalent number of normothermic patients (>36 °C) were also extracted using age, type of surgery, within the same period. (A total cohort of 212 patients). BMI was significantly higher in the normothermic patients (26.4 ± 6) compared with hypothermic patients (23.5 ± 4.1; *p* < 0.05). No other significant difference was noticed between the groups with regard of demographic and surgical characteristics.

**Figure 1:**
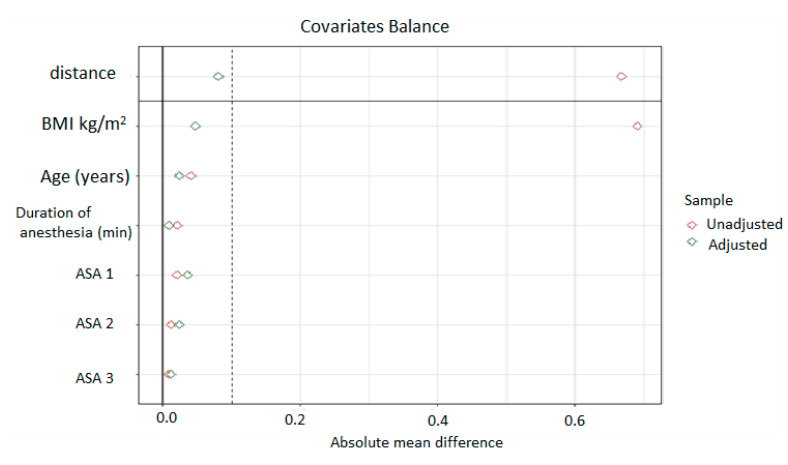
The plot represents the summary of covariate balance before and after matching. A standardized mean difference with absolute value (distance) less than 0.1 was considered an adequate reduction in the match imbalance. Dashed lines indicate standardized mean differences of −0.1 and 0.1.

After propensity score matching, 80 patients in each group were retained out of the initial 106 patients extracted (160 out of 212). The patient characteristics, monitoring status and surgical specifications for the two groups are shown in Table 1. Absolute standardized mean differences before and after matching are displayed (Figure 1, Table 1).

Surgeon’s experience (senior or junior) had no correlation with the incidence of hypothermia (*p* = 0.19). Similar results were found regarding the wound complications for each group of patients operated by the same surgeon (*p* = 0.09). The incidence of the procedures performed by junior surgeons in the study patients was 5.4%, which was not significantly different compared with all breast surgery patients (5.1%, *p* = 0.9).

**Figure 2:**
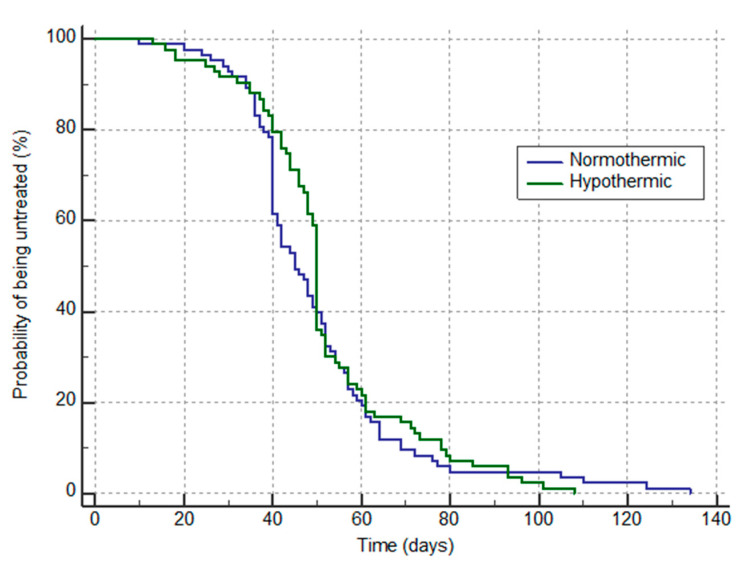
Kaplan–Meier plot of time to initiate supplemental therapy (chemotherapy or radiotherapy) in each group. The median time to initiate treatment is the time corresponding to probability of 50%. No significant difference was noticed between groups (*p* = 0.46).

The hypothermia group had significantly less monitoring and fewer active warming devices. Considering surgical sites complications, no difference was noted for other complications (Table 2). The time to initial complementary treatment (chemotherapy and/or radiotherapy) did not differ between the groups (52 ± 21 days in the hypothermia group vs. 49 ± 22 days in the control group) *p* = 0.461, Figure 2.

## 5. Discussion

This study shows the incidence of hypothermia (<36.5 °C) in breast surgery was around 27% in 2019, and the patients with hypothermia had less temperature monitoring and less active warming in this cohort of patients. The length of stay in the PACU and the occurrence of postoperative complications and time delays for adjunct treatments were not statistically different.

The incidence of inadvertent hypothermia in our group of patients undergoing breast surgery for cancer was lower in comparison to a previous multicenter study conducted in France, which reported an incidence of 53.5% for all types of surgery [15]. However, as part of our quality assurance program, we believe this incidence rate of severe hypothermia in our group of patients is high and needs to be lowered. Indeed, a large proportion of severe hypothermia patients did not have temperature monitoring and/or a warming device.

In the present study, the mean BMI was significantly lower in the hypothermia group than in the control group before matching. In fact, obesity induces physiological modifications with an increase in vasoconstriction threshold, and consequently less distribution of heat [16]. Conversely, a low BMI increases the risk of hypothermia [16].

Advanced age and an ASA class greater than 1 are known to significantly increase the occurrence of intraoperative hypothermia [17], but very few of our patients had those characteristics. No difference was noted in the remaining intraoperative parameters, although an increased risk of perioperative hypothermia has been associated with the duration of anesthesia and with surgery longer than 90 min in several studies [15,18]. 

The other objective of this study was to assess the impact of postoperative hypothermia on postoperative surgical morbidity, such as wound complications, as well as on delays in complementary oncological treatments (chemotherapy and radiotherapy). However, it should be emphasized that because of lack of power we did not want to assess any oncologic morbidity or outcome and this item was only an assessment of time delay to start potential additional oncologic treatment. Nevertheless, we found no other statistically significant morbidity among wound complications. Our initial hypothesis that perioperative hypothermia might cause a delay in the complementary oncological treatments times was also not supported by our findings. The time delay between surgery and adjunct treatments has been under extensive debate for decades, and the optimal time delay for all categories of patients has been reported [19,20,21]. Although some data exist for a specific subgroup of patients [21]. The optimal delay after surgery has been found to be about four weeks [22], which is indeed our desired delayed time; however, because of our busy chemotherapy department and the fact that some patients have their adjunct treatment in another hospital, our mean time delays (51/49 days) for both groups cannot be considered optimal. Nevertheless, we cannot speculate on this specific subject, which is beyond our expertise and the initial purpose of the present study.

Prewarming is an important measure for maintaining normothermia [23,24]; it is now part of the guidelines for preventing inadvertent perioperative hypothermia in several countries [25,26]. In addition, prewarming should be performed at least 15 min to be efficient [2,4,5,6]. We believe that prewarming was probably not performed adequately in our cohort of hypothermic patients; this might be explained by the fact that for a decade, the majority of our patients have entered the operating theaters by walking; therefore, patients cannot be prewarmed in the waiting area and they cannot be prepared, because their hospital bed is not with them. It should be noted that 15 min dedicated for prewarming per patient is a “long” time for busy and overbooked operating rooms. However, anesthesiology providers may be convinced of using this procedure if an additional item of “prewarming” alerts in our AIMS system on this issue, at least for some relevant cases as prewarming is not part of data acquisition. Other explanations could be the expected duration of short procedures such as tumorectomies, which is expected to last for less than an hour and in which only a simple blanket is used. In reality, the mean duration of surgeries was around 120 min, which itself can explain the absence of active warming or even monitoring. Nevertheless, our results indicate that temperature monitoring, as well as active air warming during the operation, reduces the risk of hypothermia, as previously described in several studies [15,27].

Cohort studies, like most observational studies, are prone to statistical bias, which could explain the lack of statistical differences found in this study for the major postoperative complications and for the time delays in complementary oncologic management. The analytical approach used in this study is relevant, given the choice of the population studied. However, the main shortcoming of this study is the size of its population, as this influenced the temperature threshold that we set and thereby directly influenced the results. We cannot exclude the possibility that, had we chosen a higher temperature threshold and generated a larger group of hypothermic patients, different results may have emerged. The process of selecting the “normothermic” control group may also have generated a risk of bias, but we tried to decrease this using a propensity score risk.

Despite the precision of the data recorded directly into the anesthetic file and the compliance (only 6% missing files), some information could still not fully reflect the reality of the practices of our anesthesia providers, such as voluntary recording of all data. In terms of quality assurance, we agree that around 33% monitoring temperature is poor, we are confident that this percentage is significantly better in major longer lasting surgery. It should also be noticed that overall mean incidence of severe hypothermia for all of our patients was around 1%. Nevertheless, this study permits us to focus on monitoring temperature for expected short procedures and provide data to improve this shortcoming for future assessment.

## 6. Conclusions

In this propensity score-matched study, severe hypothermia of less than 32.5 °C was associated with the lack of intraoperative monitoring and active warming in breast surgery cancer patients. Due to insufficient power, we could not detect a significant difference in wound complications and delays in adjunct oncological treatment. 

## Figures and Tables

**Table 1:** Patient characteristics comparison after matching.

	Group Control *n* = 80	Group Hypothermia *n* = 80	*p* Value
Age (year), mean (SD)	58.95 (11.56)	59.25 (12.87)	0.87
Sex ratio (F/M) %	83/17	82/18	1
BMI (kg/m^2^), mean ± SD	24.34 ± 4.01	21.4 ± 6.1	0.756
ASA (1/2/3/) (*n*)	16/60/7	13/62/8	0.81
Tumorectomy +/− Lymph nodes (*n*)	29	27	
Mastectomy +/− lymph nodes resection (excluding axillary) (*n*)	19	20	
Axillary nodes resection/tumorectomy redux (*n*)	20	18	
Tumor resection + axillary nodes + oncoplasty/Mastectomy and axillary nodes (*n*)	15	18	0.923
Intraoperative temperature monitoring (*n*)	23 (27.7)	11 (13.3)	0.03
Active warming (*n*)	27 (32.5)	11 (13.2)	0.006
Simple blanket (*n*) Duration of surgery (mean SD)	7 (9) 135(58)	7 (8) 134(57)	1 0.9
Temperature at arrival PACU (mean SD)	36.81 (0.29)	35.01 (0.2)	<0.001
Duration of PACU min mean (SD)	89 (31)	91 (41)	0.7

BMI: body mass index; ASA: American Society of Anesthesiologists physical status, SD: standard deviation.

**Table 2:** Surgical sites complications.

	Group Control *n* = 80	Group Hypothermia *n* = 80	Risk	*p* Value
Infection (*n*; %)	6; 7.5	7; 8	1.16 (0.41–3.31)	0.77
Cutaneous Necrosis (*n*; %)	0; 0	4; 4	9 (0.49–164)	0.14
Hemorrhage (*n*; %)	7; 6.6	8; 10	1.14 (0.43–3)	0.78
Lymphocele (*n*; %)	20; 25	18; 22	0.9 (0.51–1.56)	0.71

Odds ratio for all surgical site complications: 1.73, 95% CI 0.85–3.23, *p* = 0.08.

## Data Availability

The data presented in this study are available on request from the corresponding author.
